# Supplementary material for: Seeing Beyond RGB Capabilities: Data-Driven and Physics-Guided Broadband Spectral Extrapolation of Plasmonic Nanostructures by Deep Learning
Source: ACS Photonics. 2026 Apr 28;13(16):4434–48. doi: 10.1021/acsphotonics.5c02828 (PMC13495945; doi:10.1021/acsphotonics.5c02828)
Supplement: Supplementary file 1 [file ph5c02828_si_001.pdf]

Supporting Information: Seeing Beyond RGB Capabilities: Data-Driven and Physics-Guided Broadband Spectral Extrapolation of Plasmonic Nanostructures by Deep Learning

Mohammadrahim Kazemzadeh,[†,#] Banghuan Zhang,[‡,#] Tao He,[‡] Haoran Liu,[‡] Zihe Jiang,[‡] Zhiwei Hu,[‡] Xiaohui Dong,[‡] Chaowei Sun,[¶] Wei Jiang,[¶] Xiaobo He,[§] Shuyan Li,[‖,⊥] Gonzalo Álvarez-Pérez,[†] Ferruccio Pisanello,[†] Huatian Hu,[*,†,@] Wen Chen,[*,‡] and Hongxing Xu[*,‡,§,¶]

†*Center for Biomolecular Nanotechnologies, Istituto Italiano di Tecnologia, via Barsanti 14, Arnesano, 73010, Italy.*

‡*State Key Laboratory of Precision Spectroscopy, East China Normal University, Shanghai, 200241, China*

¶*Institute of Laser Manufacturing, Henan Academy of Sciences, Zhengzhou, 450046, China*

§*Institute of Physics, Henan Academy of Sciences, Zhengzhou, 450046, China*

‖*Tsinghua Shenzhen International Graduate School, Tsinghua University, Shenzhen, 518055, China.*

⊥*School of EEECS, Queen's University Belfast, Belfast, Northern Ireland, BT9 5BN, United Kingdom*

#*These authors contributed equally to this work*

@*Current address: POLIMA—Center for Polariton-driven Light–Matter Interactions, University of Southern Denmark, Odense M, Denmark*

E-mail: huhu@mci.sdu.dk; wchen@lps.ecnu.edu.cn; hxxu@hnas.ac.cn

Contents

S1 Comparison of DF images and spectra of DL selections **S3**

S2 Unsupervised UMAP analysis of NPoMs **S4**

S3 SPARX Model Architecture **S5**

S4 Homoscedastic *SPARX* Model Performance Evaluation **S7**

S5 Testing the generality of *SPARX*: plasmonic nanocube-on-mirror (NCoM) cavities **S12**

S6 Shape Classification from DF RGB Images: Unsupervised Study **S14**

S7 Distinguishability of Visually Similar Dark-Field Images in High-Dimensional Feature Space **S16**

References **S20**

20 pages, 10 figures in total.

S1 Comparison of DF images and spectra of DL selections

Figure S1 shows the comparison of the DF images selected by DL (main text Figure 1) with their corresponding spectrum. The DF images with yellow hues tend to have a more redshifted higher-order peak or shoulder than the DF images with green hues. Taking the DF image with index = 149 or 785 for example, the hues are green due to the minor peaks near 580 nm. While the index = 646 case shows a more yellow-ish hue due to the slightly redshifted peak around 620 nm. However, from the scattering spectra, all of these nine cases have very similar main peaks around 820 nm.

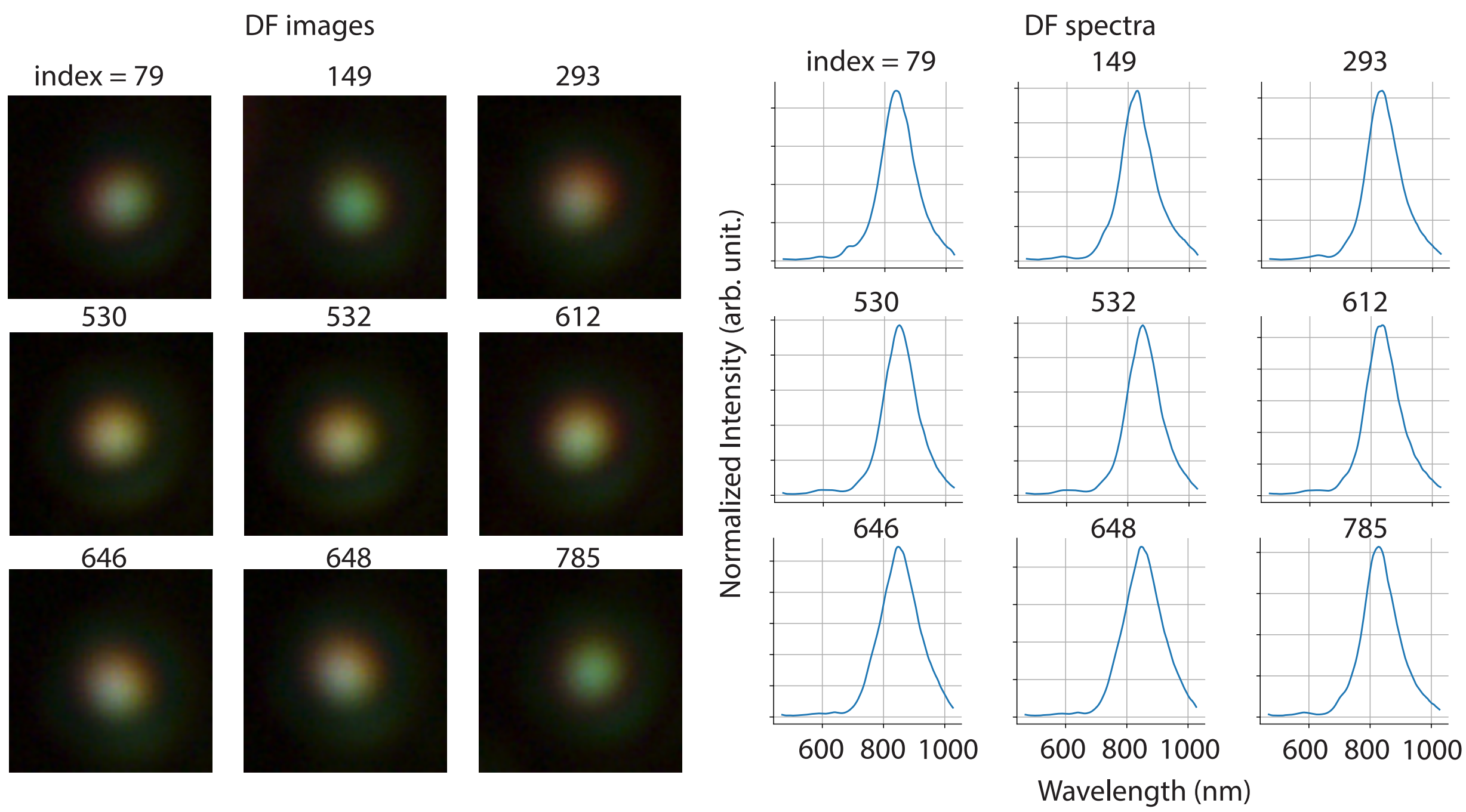

Figure S1: Comparison of the DF images and the spectra of DL selection in the main text Fig. 1.

S2 Unsupervised UMAP analysis of NPoMs

In contrast of PCA analysis in the main text Fig. 2, we did UMAP analysis in Fig. S2. Spectral data were embedded in one dimension (Fig. S2(a)) and two dimensions (Fig. S2(b)), while DF images were projected in two dimensions S2(c)). Coloring the DF RGB images UMAP projection by spectral-derived features—including spectral UMAP values, peak positions, and total spectral energy—revealed clear visual correlations between the DF and spectral modalities.

We also performed 2D UMAP on the spectra (Fig. S2(b)). Similar to their 1D embedding (Fig. S2(a)), the 2D UMAP of the spectra does not show clear clustering (Fig. S2(a)). However, when color-coded by the first UMAP component of the DF RGB images (Fig. S2(b)), a clear gradient emerges, indicating a correlation between spectral features and DF scattering patterns. In contrast, the 2D UMAP of the DF RGB images exhibits clear clustering (Fig. S2(c)).

These observations suggest that DF RGB images contain additional information beyond spectral features alone, as expected since the DF scattering (Airy) pattern reflects both near-field and far-field nanoparticle responses.

The DF UMAP projection displays multiple distinct clusters, in contrast to the relatively continuous distribution observed in PCA projections. These clusters correspond closely to specific spectral characteristics. For example, the cluster in the lower-right region exhibits a color gradient where resonance wavelength and total spectral energy vary inversely—i.e., as the resonance red-shifts, intensity decreases. Coloring by sample index, which reflects acquisition time, shows no systematic trend, confirming high reproducibility and stability of measurements across multiple days.

Mapping spectral features, such as peak position and total energy, onto the DF UMAP space highlights the rich spectral information inherently encoded in the images, which is typically inaccessible in conventional DF image analysis. The nonlinear nature of UMAP produces more distinct clusters than PCA, providing an intuitive visualization of the com-

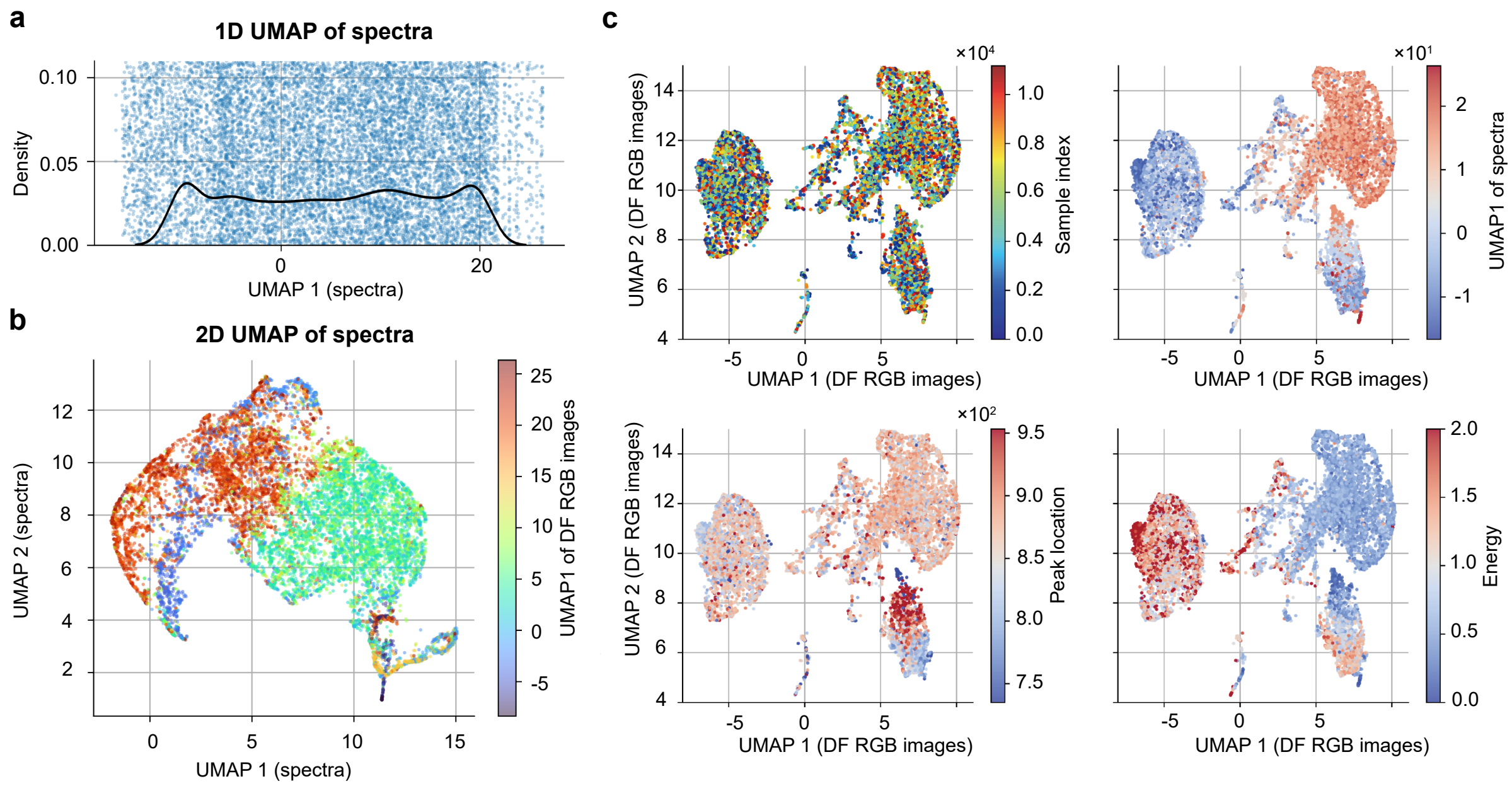

Figure S2: **Unsupervised learning (UMAP) of the NPoM's dark-field information.** 1D (a) and 2D (b, c) UMAP projections showing correlations between DF RGB images and spectral data, with spectra projections (b) colored by the UMAP1 of DF RGB, DF RGB images projections (c) colored by the index of the samples (timeline), UMAP of spectra, peak location (λ), and integrated energy.

plex relationship between DF images and spectral properties. These observations indicate that unsupervised UMAP analysis robustly captures the strong DF–spectra correlation, supporting subsequent supervised modeling for spectral prediction.

S3 SPARX Model Architecture

We propose an autoencoder-like architecture,[1?] as illustrated in Figure S3. The *SPARX* consists of an encoder that compresses DF images into a latent space using a series of 2D convolutional layers. To mitigate issues such as vanishing gradients in deep networks, we employ residual connections[2] within cascaded 2D convolutional layers, as depicted in the 2D convolutional operator block. Each convolutional layer is followed by batch normalization[3] and a nonlinear ReLU activation function. Additionally, max pooling is used to progressively reduce the feature map size. The input tensor, originally shaped as (128, 128, 3), where

(128, 128) represents the spatial dimensions and 3 represents the color channels (RGB), is gradually reduced in size. Eventually, it is compressed into a compact representation of (4, 4, 512), where (4, 4) represents the reduced spatial dimensions and 512 represents the feature channels extracted by the convolutional layers. This tensor is then reshaped into a single dense layer with a shape of (8192), which is further reduced to (4096) using a fully connected layer with linear activation. Since convolutional layers primarily preserve local features, this final dense layer enhances the ability to capture non-local correlations, ensuring that the encoder produces a representation suitable for the decoder.

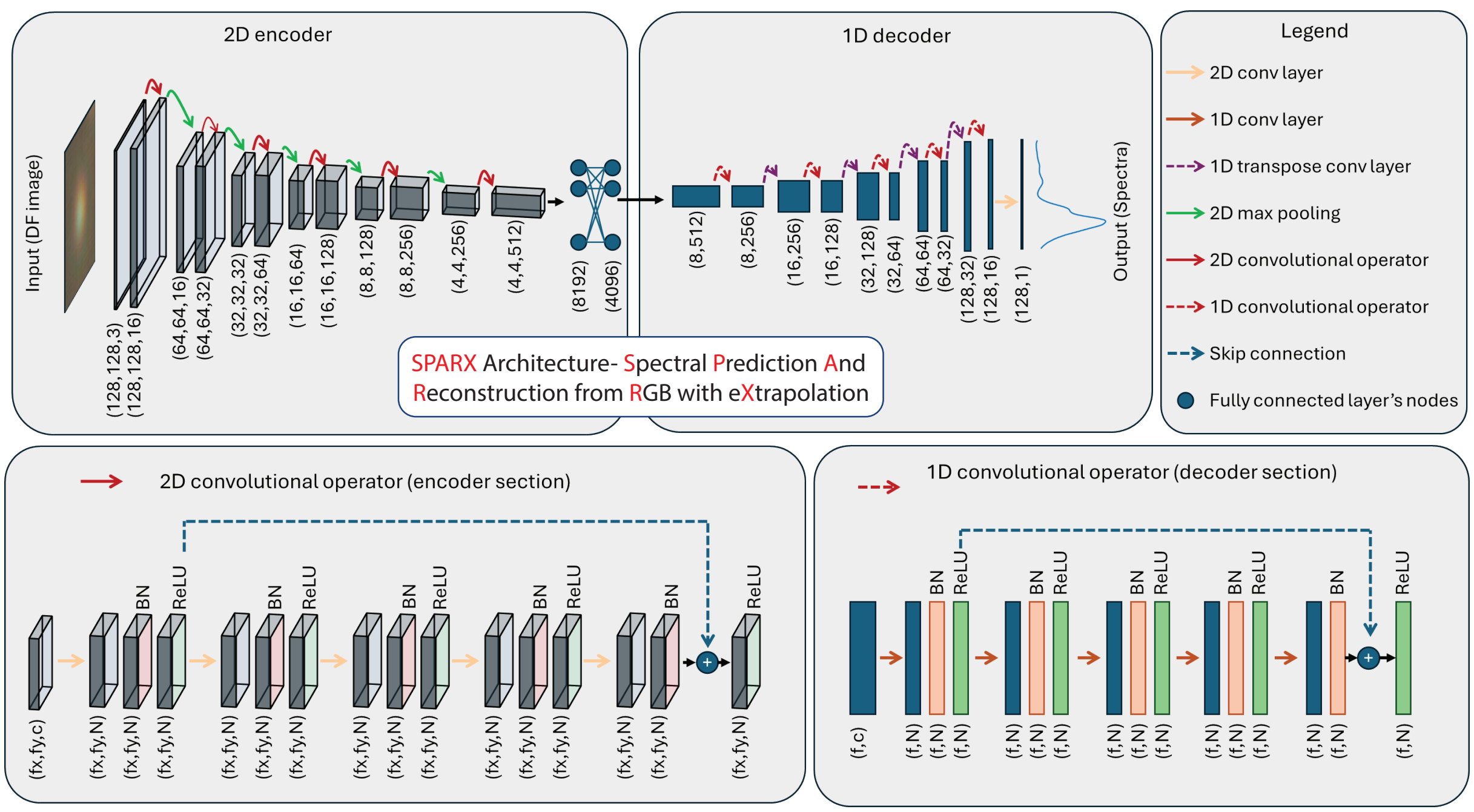

Figure S3: **The architecture of *SPARX*.** Autoencoder architecture for translating DF images (2D) to spectra (1D). The encoder uses 2D convolutions with residual connections, and the decoder reconstructs spectra with 1D convolutions. Skip connections prevent gradient vanishing.

The decoder starts by reshaping the (4096) latent vector into (8, 512), where 8 represents the spatial length, and 512 represents the feature channels. A series of 1D convolutional layers with batch normalization and ReLU activation functions are then applied, as illustrated in the 1D convolutional operator. Similar to the encoder, we incorporate skip connections to prevent gradient vanishing. The feature size is gradually increased to (128,16), where 128

corresponds to the spectral resolution and 16 represents the feature channels. Finally, a single 1D convolutional layer is used to map the output to the spectral shape of (128,1), where 128 represents the spectral resolution and 1 denotes the single spectral intensity value for each wavelength. To prevent overfitting, we employ a combination of L1 and L2 regularization across all convolutional layers, with regularization coefficients of $\alpha = 10^{-5}$ for L1 and $\beta = 10^{-4}$ for L2.

S4 Homoscedastic *SPARX* Model Performance Evaluation

For the first try, we begin by evaluating the error of the *SPARX*'s spectral reconstruction. A baseline comparison is made using the average spectrum of all the particles. The reason for using the average as the reference point is that, in the absence of a better method, statistically, the average spectrum provides the best guess for the spectra. In Figure S4 (a), We show the MAE of the spectra when the average spectrum is used as the prediction and the DL output is shown in orange and blue, respectively, across the wavelength range. The formula for MAE is given as:

$$\text{MAE}(\lambda) = \frac{1}{N}\sum_{i=1}^{N} |y_i(\lambda) - \hat{y}_i(\lambda)| \tag{1}$$

where N is the total number of samples in the testing set, $y_i(\lambda)$ is the ith ground truth spectrum at wavelength λ, and $\hat{y}_i(\lambda)$ is the ith predicted spectrum at wavelength λ.

The spectral range that each color of the camera is capturing is also shown in Figure S4(a) as shades on the background. The key point here is that while the camera captures data only up to 700 nm, the major (in terms of energy of the spectrum) part of the spectral reconstruction happens outside this range. This demonstrates that the DL model can extrapolate information beyond the given spectral range (DF image). Additionally, The MAE

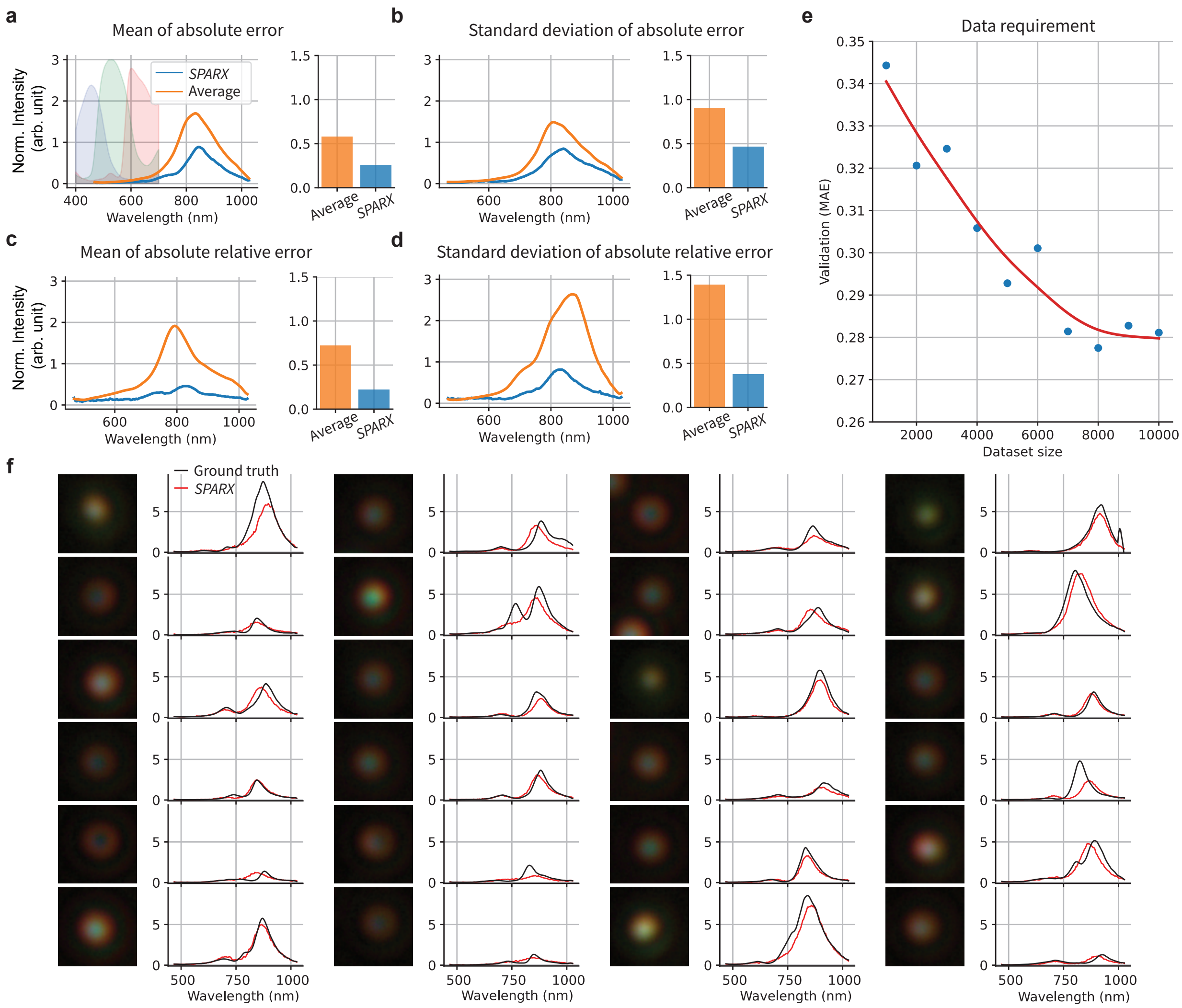

Figure S4: **Error analysis of spectral reconstruction by the homoscedastic *SPARX*.** (a) Mean absolute error (MAE) of the predicted spectra compared to the average spectra, showing the camera's spectral range and extrapolation beyond 700 nm. (b) Standard deviation of the reconstruction error across wavelengths. (c) Mean absolute relative error (MARE) showing more consistent error distribution. (d) Standard deviation of the absolute relative error. (e) Performance improvement with increasing training data size. (f) Example data reconstructions with corresponding ground truth and DF images.

is presented as a bar chart in Figure S4(a) for comparison. The standard deviation of the error across all wavelengths is calculated and shown in Figure S4(b). It is defined as:

$$\sigma_{\text{error}}(\lambda) = \sqrt{\frac{1}{N}\sum_{i=1}^{N}\left(|y_i(\lambda) - \hat{y}_i(\lambda)| - \text{MAE}(\lambda)\right)^2} \tag{2}$$

Moreover, to assess the performance more fairly, we introduce the Mean Absolute Relative Error (MARE), as shown in Figure S4 (c), across the wavelength. The formula for MARE is defined as:

$$\text{MARE}(\lambda) = \frac{1}{N}\sum_{i=1}^{N}\left|\frac{y_i(\lambda) - \hat{y}_i(\lambda)}{y_i(\lambda) + \epsilon}\right| \tag{3}$$

where ϵ is a small constant value ($\epsilon = 1.2{\times}10^{-1}$ for this problem) added to the denominator to prevent division by small or negative numbers, which could arise due to smoothing operations that reject the noise in spectroscopy measurements.

Additionally, we calculate the standard deviation of the absolute relative error, which is shown in Figure S4 (d). The formula for this error metric is defined as:

$$\sigma_{\text{MARE}}(\lambda) = \sqrt{\frac{1}{N}\sum_{i=1}^{N}\left(\left|\frac{y_i(\lambda) - \hat{y}_i(\lambda)}{y_i(\lambda) + \epsilon}\right| - \text{MARE}(\lambda)\right)^2} \tag{4}$$

where MARE(λ) is the Mean Absolute Relative Error calculated previously for each wavelength λ. The metric introduced to evaluate the DL performance, for both Figures S4 (c) and (d), shows that the DL predictions are more consistent across the wavelengths. In particular, the MAE shows a prominent peak around 800 nm, which is expected due to the higher intensity of the spectral data in this range. This higher intensity leads to larger errors, especially around the 800 nm region. However, this peak is not as prominent in the MARE, indicating that the MARE provides a more balanced measure of error across the spectral range. This suggests that the *SPARX* model presents ~3 times better prediction of both the intensity and variation of the spectra in terms of MARE and σ_{MARE} metrics, respectively.

Another important observation is that the standard deviation (σ_{MARE} and σ_{MAE}) varies across different wavelengths, which suggests that the error is not constant but instead increases or decreases depending on the wavelength. This phenomenon indicates that the problem is heteroscedastic, and as such, the model's prediction uncertainty is wavelength-dependent. This variation in error distribution calls for a more sophisticated loss function that can account for this non-homogeneous nature of the data, thereby improving the model's performance.

Finally, we perform an analysis on how many data points are needed for effective training of the model. This is done by gradually increasing the size of the dataset and evaluating the model's performance over a validation set. The result is shown in Figure S4 (e), where we observe that performance improvement tapers off after around 7,000 data points. Few randomly selected examples of the data reconstruction are also shown in Figure S4 (f), along with their corresponding ground truth and DF images. All the spectra are plotted in the same y-axis with the same normalized intensity scale, Figure S4(f) clearly shows that our DL model can reliably capture both the intensity and the lineshape of the spectrum from the DF images inputs. Furthermore, while the trained *SPARX* model cannot achieve perfect spectral reconstruction due to the inferential nature of the task, this stems from the limited far-field and geometric information in DF images. The inherent randomness of nanoscale structures adds further complexity, highlighting the need for sophisticated methods to manage uncertainty from incomplete measurements.

In Figure S5(a), we show the peak wavelength error ($\Delta\lambda$) and peak intensity error (ΔI) when using the average spectrum over all 1,000 test data points as a prediction, compared against each individual ground truth spectrum. Figure S5 (b) shows the same analysis but from the *SPARX* model applied to the whole test dataset. These 2D joint plots are accompanied by marginal histograms along each axis, illustrating the population distribution of $\Delta\lambda$ and ΔI. Besides the widespread distribution, a key observation in Figure S5 (a) is the presence of structured branches, indicating a correlation between spectral peak intensity and

error in resonance. This structure disappears in Figure S5 (b), suggesting that the trained *SPARX* model has effectively learned these nonlinear relationships and integrated them into the spectral reconstruction.

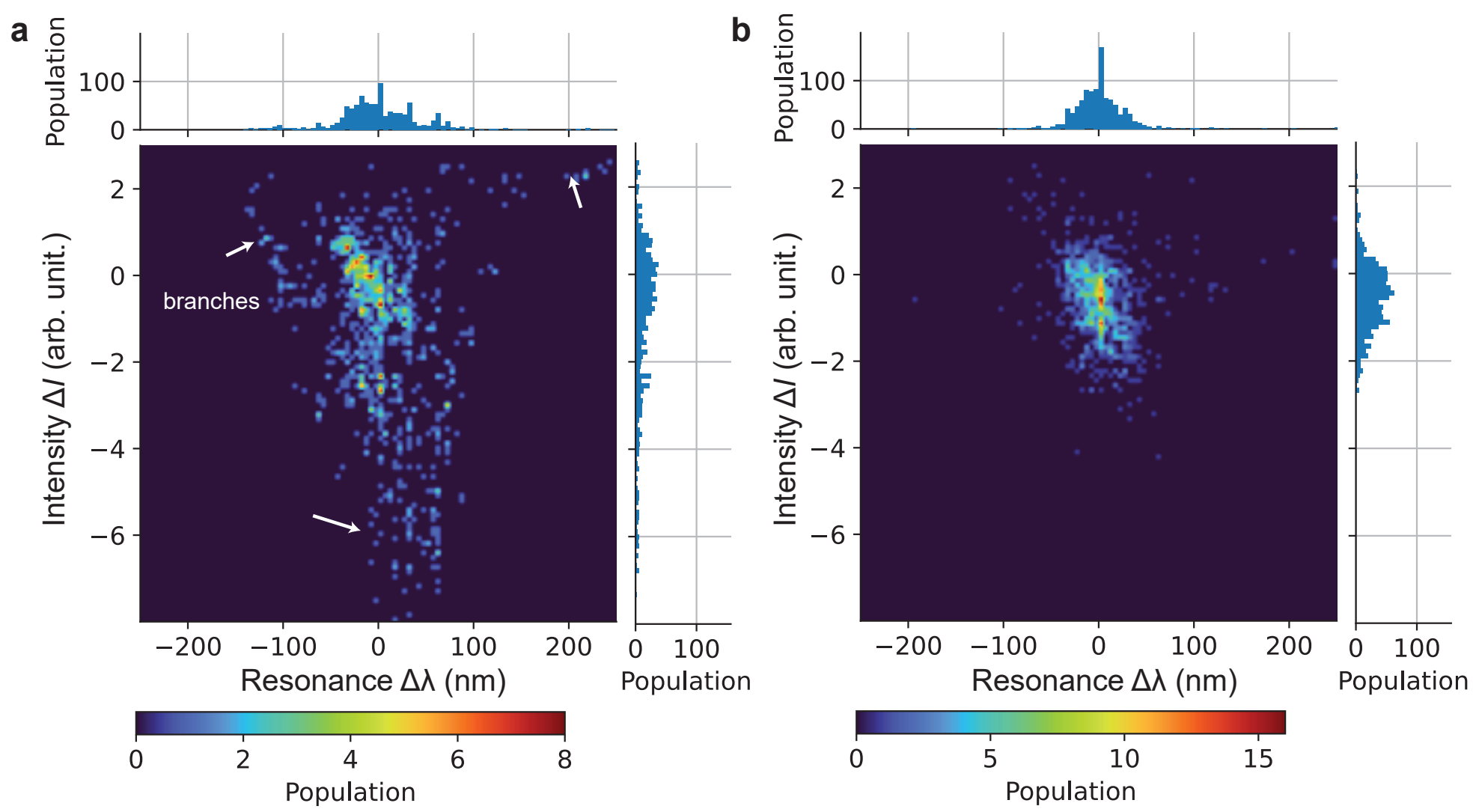

Figure S5: **Joint Distribution** (a) Comparison of errors of spectral resonance peak ($\Delta\lambda$) and intensity differences (ΔI) using (a) the average spectrum as a predictor and (b) the *SPARX* model. Joint 2D histograms show that the *SPARX* model learns nonlinear correlations, reducing prediction errors and producing a more Gaussian-like distribution.

Furthermore, the population distributions of both intensity and wavelength differences are significantly narrower in the *SPARX* predictions compared to using the average spectrum. The distributions in Figure S5 (b) appear Gaussian, which may be attributed to the intrinsic uncertainties that cannot be resolved using only RGB data, given that the camera captures spectral information only up to 700 nm. From these Gaussian distributions, *SPARX* prediction has made a considerable step forward compared to the statistical prediction. To be fair, the population distribution of the average-spectrum "prediction" in Figure S5(a) merely reflects the geometric consistency across the NPoM systems, rather than constituting a true predictive method, as it lacks the capability to identify or filter individual nanoparticles with desired spectral responses. Instead, it serves only as a statistical baseline. However, *SPARX* overcomes this limitation by accurately predicting individual spectra that match the ground truth obtained from spectroscopy (as evidenced by small $\Delta\lambda$ and low ΔI of Figure S5(b)),

thereby offering a practical route to circumvent the inherent spectral variations in plasmonic systems.

S5 Testing the generality of *SPARX*: plasmonic nanocube-on-mirror (NCoM) cavities

To further demonstrate the generality of our *SPARX* model beyond spherical NPoM nanogaps, we evaluate it on an additional dataset consisting of cubic nanoparticles (i.e., nanocube-on-mirror nanogaps, NCoMs). Figure S6 provides additional details on the spectral reconstruction performance for the NCoM nanocavities. In this example, we used the same optical setup and spectral acquisition parameters as in the nanosphere case.

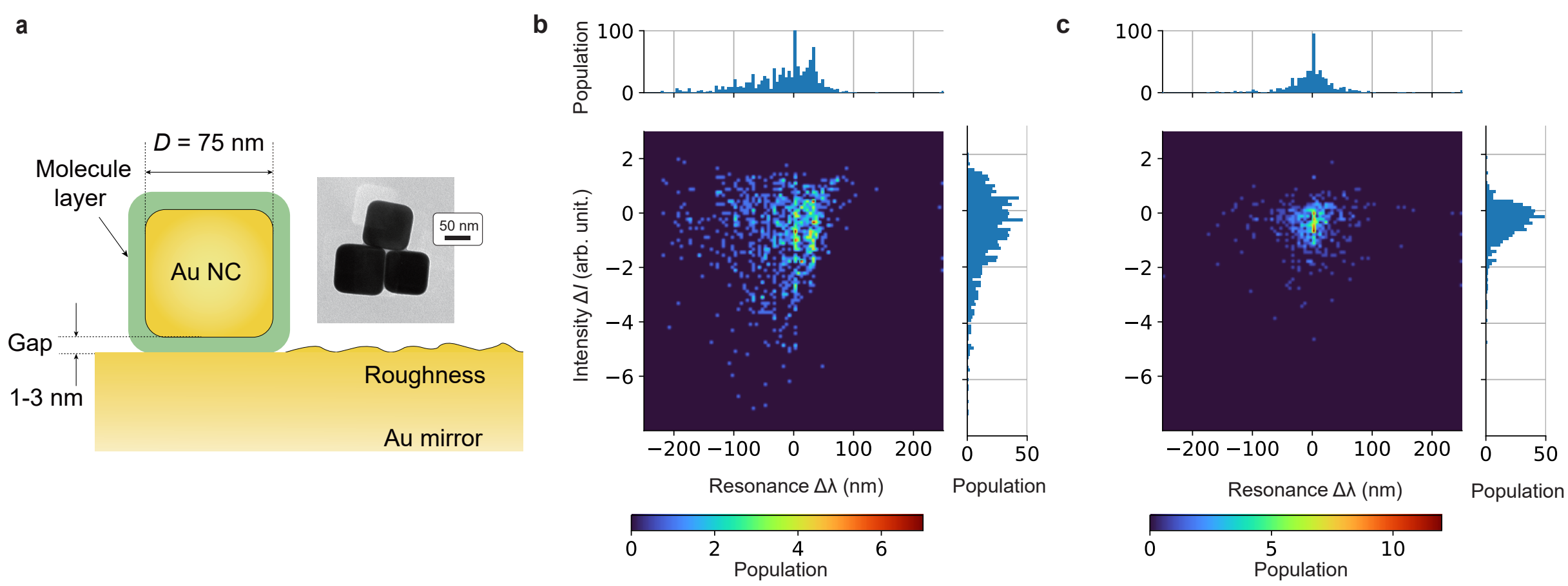

Figure S6: **Joint Distribution of plasmonic (nanocube) NCoM nanocavities** (a) Comparison of errors of spectral resonance peak ($\Delta\lambda$) and intensity differences (ΔI) using (a) the average spectrum as a predictor and (b) the *SPARX* model. Joint 2D histograms show that the *SPARX* model learns nonlinear correlations, reducing prediction errors and producing a more Gaussian-like distribution.

Panel Fig. S6(a) shows the designed geometry and measured average dimensions of the nanocube, together with TEM images illustrating the variability in edge sharpness and facet morphology typical of the synthesis methods. The nanocubes with an average size of 75 nm were bought from MeLab. These geometric variations are one of the primary contributors

to the observed heterogeneity in optical response (see the widely spread differences in the resonance peak locations and intensity in Fig. S6(b)).

Fig. S6 (b) compares the error in the prediction of the strongest resonance with that of a naive baseline estimate (mean of all spectra). The error spread is noticeably broader than in the nanosphere case, consistent with the larger spectral diversity of the NCoM dataset. This diversity arises not only from size variation but also from subtle differences in cube orientation and gap morphology, which can introduce multiple closely spaced resonances. Fig. S6 (c) demonstrates that SPARX is able to capture these complex spectral signatures far more effectively than the naive approach, substantially reducing the reconstruction error even for highly heterogeneous samples. Interestingly, the compression performance for NCoMs in Fig. S6 (c) exhibits an even clearer trend than in the NPoM systems, as naive guesses from statistic baseline (i.e., the average) for NPoMs benefit from the greater similarity among cases. This underscores the higher complexity of NCoMs and highlights the *SPARX* model's ability to tackle such challenging data far beyond the reach of naive statistical baselines.

Quantitative Error Metrics. While Fig. S6 highlights representative error distributions, Fig. S7 presents a comprehensive set of quantitative metrics for the homoscedastic *SPARX* model applied to NCoM spectra similar to the ones in Fig. S4. The MAE plot in Fig. S7(a) shows that prediction errors remain low across most of the spectral range and that the model successfully extrapolates beyond the 700 nm detection limit of the camera. Fig. S7(b) provides the standard deviation of reconstruction error across wavelengths, revealing slightly higher variability near plasmonic resonance peaks, where sensitivity to geometric perturbations is greatest. The MARE in Fig. S7(c) confirms that the relative error remains consistently low across the spectrum, while Fig. S7(d) shows that the standard deviation of MARE is similarly stable, indicating robust performance even for particles whose spectral features deviate significantly from the mean. These complementary metrics strengthen the conclusion that *SPARX* is well-suited for reconstructing spectra from minimal RGB information in NCoM systems.

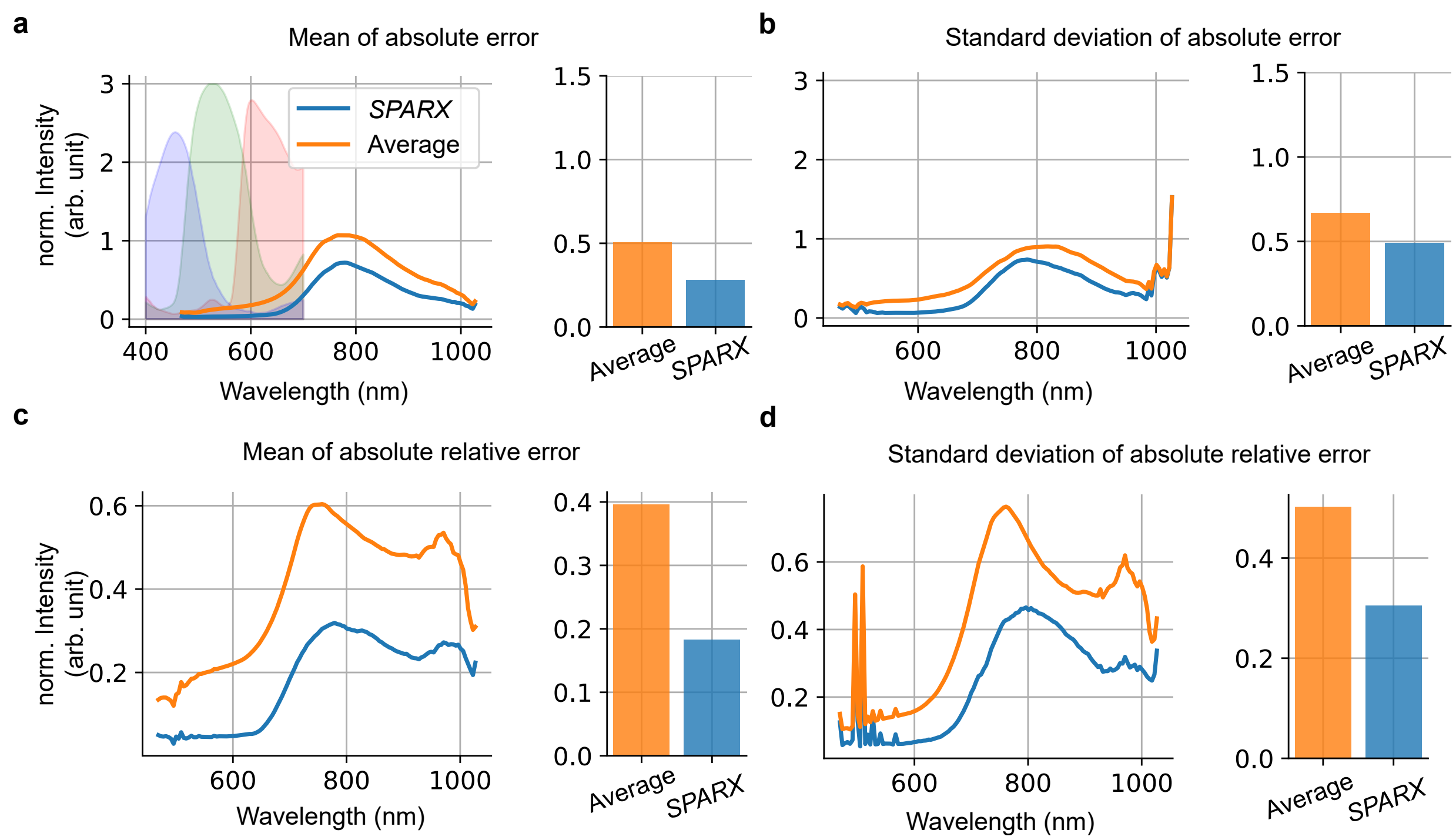

Figure S7: **Error analysis of NCoM's spectral reconstruction by the homoscedastic *SPARX*.** (a) Mean absolute error (MAE) of the predicted spectra compared to the average spectra, showing the camera's spectral range and extrapolation beyond 700 nm. (b) Standard deviation of the reconstruction error across wavelengths. (c) Mean absolute relative error (MARE) showing more consistent error distribution. (d) Standard deviation of the absolute relative error.

S6 Shape Classification from DF RGB Images: Unsupervised Study

While fast and precise spectral prediction can be achieved using alternative novel spectroscopic methods, such as hyperspectral cameras, a machine-learning-based model offers capabilities that extend beyond this task (i.e., acquiring the spectra). Here, we demonstrate an application for classifying nanoparticle shapes from individual DF images, without the need to acquire their spectra.

Figure S8 provides supplementary unsupervised analysis aimed at assessing whether DF RGB images contain enough information to differentiate nanocubes from nanospheres without supervised labels. The normalized images in Fig. S8 (a) demonstrate the removal of

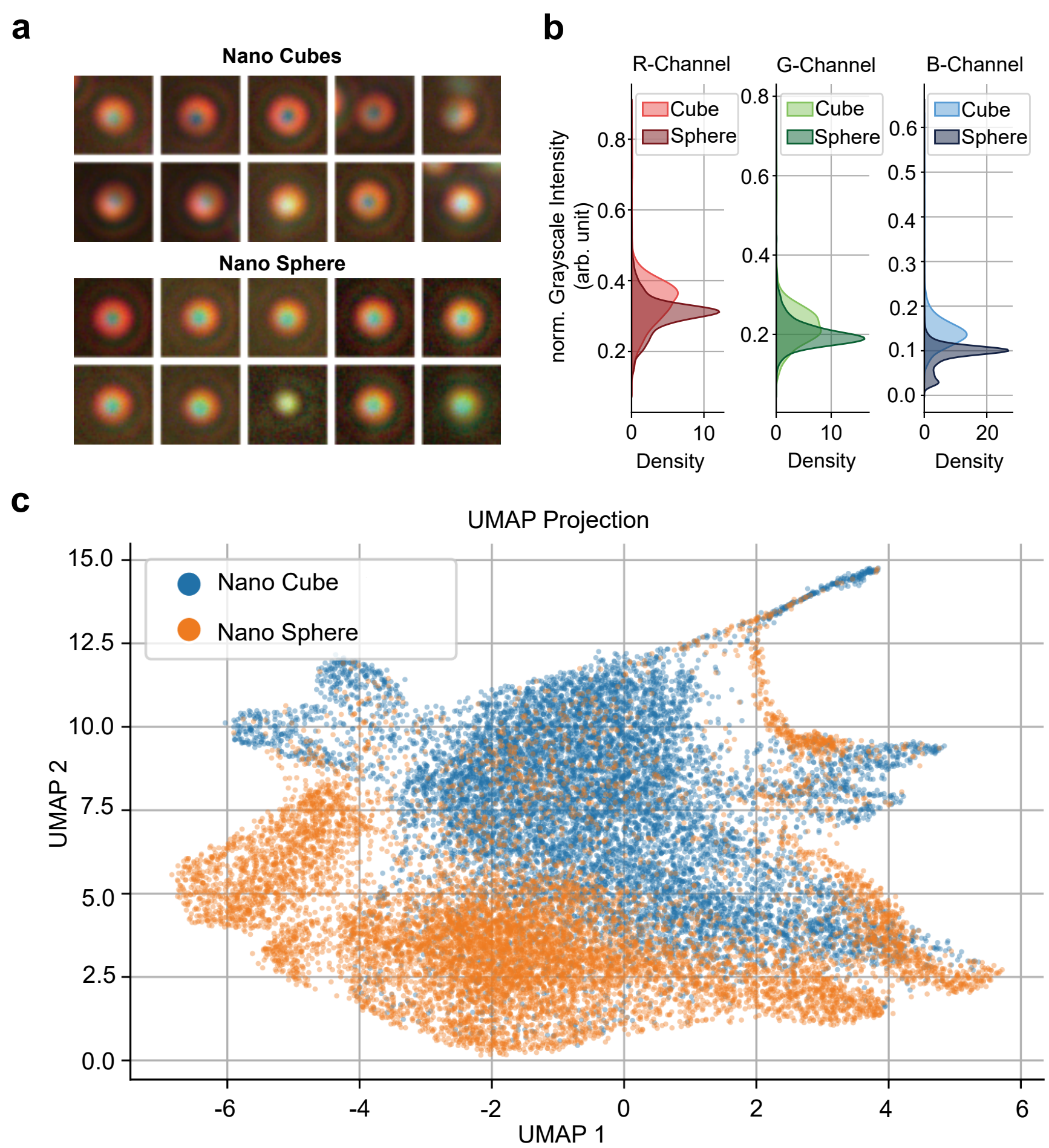

Figure S8: **Normalization and unsupervised study of nanocube and nanosphere DF images** (a) Examples of normalized DF RGB images for both nanospheres and nanocubes. (b) Intensity distributions for each RGB channel. (c) UMAP projection of the normalized RGB data

acquisition-dependent brightness variations, ensuring that observed differences are intrinsic to the scattering patterns. The channel-wise intensity histograms in Fig. S8 (b) reveal that nanospheres exhibit narrower KDEs with peaks at lower intensities, reflecting their more uniform scattering distribution. Nanocubes, by contrast, produce broader distributions, consistent with their more complex near-field patterns and multiple resonance modes.

Finally, the UMAP projection in Fig. S8 (c) shows partial but distinct grouping of nanosphere and nanocube data points. This separation suggests that DF RGB images inherently encode structural information, thereby motivating the use of supervised learning for precise classification.

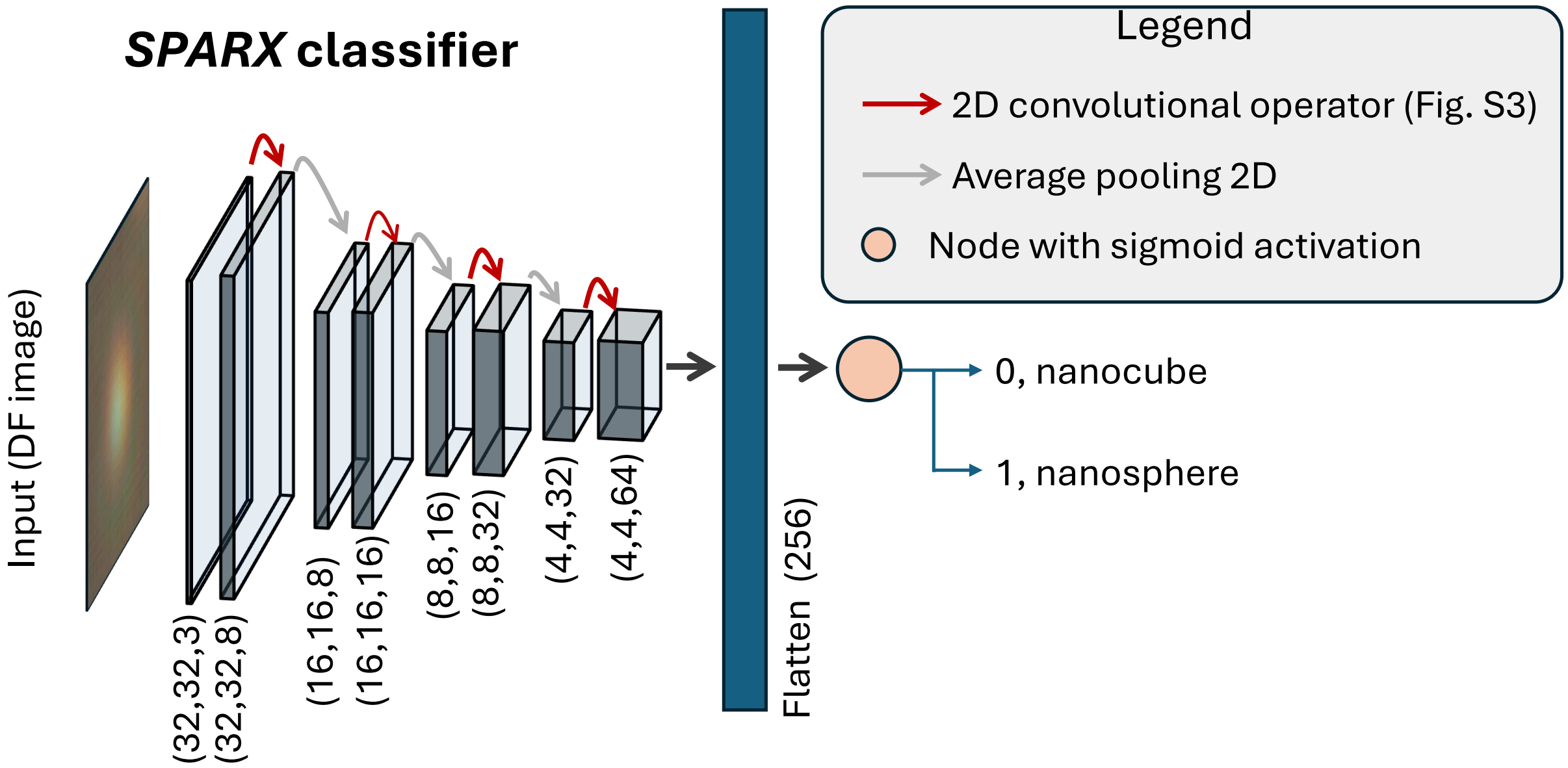

Figure S9: **The architecture of *SPARX* classifier.**

The architecture of the *SPARX* classifier is shown in Fig. S9.

S7 Distinguishability of Visually Similar Dark-Field Images in High-Dimensional Feature Space

A potential concern when predicting spectra from RGB dark-field images is the apparent "non-uniqueness" of the mapping: different spectral lineshapes could in principle produce similar integrated RGB colors within the limited < 700 nm detection range. However, dark-field images that appear visually similar are not equivalent in their pixel-level numerical representation or in high-dimensional feature space.

First, in practice, dark-field images that appear visually similar are not identical in the high-dimensional pixel space used by the model. Due to variations in angular scattering distributions and modal interference, nanoparticles with similar overall RGB appearance exhibit subtle but systematic differences in pixel intensity distributions and spatial textures. Secondly, all those resonances appeared in the spectra are physically correlated since they

obey the Mie theory and plasmon hybridization rule. Last but not least, plasmons are in general lossy and the tails of the fundamental modes often overlap with the RGB channel. And for the extreme cases that the systems work in the infrared to the mid-infrared regimes, the users are suggested to add additional channel in this specific wavelength to capture the signals. But overall, the SPARX is a general framework.

To quantitatively illustrate the differences of the RGB information-limited dark-field images, a pair of dark-field images with highly similar RGB appearances was selected (Fig. S10a). The pair was first screened by visual inspection and then validated using quantitative similarity metrics. Their Euclidean distance in high-dimensional pixel space falls within the bottom 5% of the pairwise distance distribution across the validation set, while their cosine similarity ranks within the top 8%, indicating statistically high similarity. For pixel-level comparison, the spot centers were determined via Gaussian fitting, and one image was translationally aligned to the other. Channel-wise difference maps for R, G, and B were then computed (Fig. S10b). The resulting maps show non-zero, spatially structured residuals, indicating that subtle but systematic pixel-level differences remain between the two images.

Further comparison was performed using RGB histograms of the same image pair (Fig. S10c). Although the differences are imperceptible to human vision and the pixel values are numerically very close, the histogram distributions do not completely overlap. Quantifiable discrepancies can be observed in peak positions, distribution widths, and tail profiles, statistically confirming that the two images are not numerically equivalent in feature space.

To further demonstrate the spectral implications of these subtle image differences, several groups of visually similar dark-field image pairs were analyzed (Fig. S10d). Each pair was validated by Euclidean distance falling within the bottom 5% of the pairwise distance distribution across the validation set. Despite their high macroscopic visual similarity, the corresponding experimental scattering spectra differ substantially. The *SPARX* model accurately captures these subtle yet physically meaningful high-dimensional differences and successfully predicts the distinct spectral lineshapes.

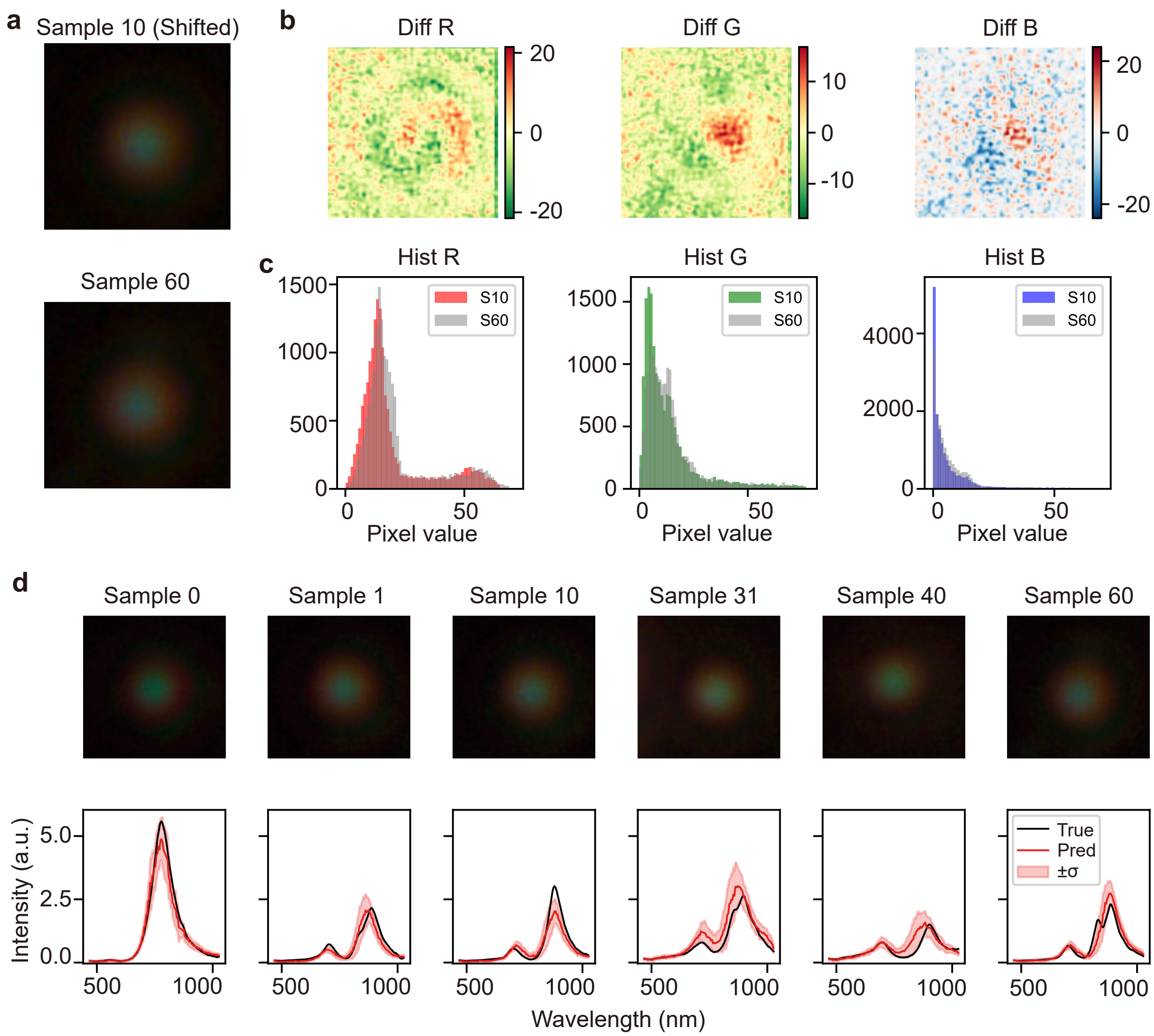

Figure S10: **The model captures subtle differences beyond human visual perception.** (a) Two dark-field images manually selected for their high visual similarity. Sample 10 was translationally aligned to Sample 60 by locating the spot center via Gaussian fitting and applying a corresponding shift. (b) Channel-wise difference maps (R, G, and B) between the two images, revealing non-zero and spatially structured residuals.(c) RGB histogram comparisons of the two images, demonstrating measurable distributional discrepancies despite their similar visual appearance.(d) A set of visually similar dark-field images together with their corresponding experimental spectra and SPARX predictions, showing that although the images appear alike to the human eye, their spectral responses differ substantially, and the model accurately resolves and predicts these differences.

These observations indicate that visually similar RGB images remain distinguishable in the high-dimensional feature space used by the model. As a result, the model does not rely solely on coarse color information but instead utilizes subtle spatial and statistical features embedded in the images to infer the underlying spectral response.

References

(1) Kazemzadeh, M.; Martinez-Calderon, M.; Xu, W.; Chamley, L. W.; Hisey, C. L.; Broderick, N. G. Cascaded deep convolutional neural networks as improved methods of preprocessing raman spectroscopy data. *Analytical Chemistry* **2022**, *94*, 12907–12918.

(2) He, K.; Zhang, X.; Ren, S.; Sun, J. Deep residual learning for image recognition. Proceedings of the IEEE conference on computer vision and pattern recognition. 2016; pp 770–778.

(3) Ioffe, S.; Szegedy, C. Batch normalization: Accelerating deep network training by reducing internal covariate shift. International conference on machine learning. 2015; pp 448–456.
